# Healthcare Facilities as Potential Reservoirs of Antimicrobial Resistant *Klebsiella pneumoniae*: An Emerging Concern to Public Health in Bangladesh

**DOI:** 10.3390/ph15091116

**Published:** 2022-09-07

**Authors:** Zahid Hayat Mahmud, Salman Zahir Uddin, M. Moniruzzaman, Sobur Ali, Monir Hossain, Md. Tamzid Islam, Dorin Teresa D. Costa, Mohammad Rafiqul Islam, Md. Shafiqul Islam, Md. Zakiul Hassan, Li-Ann Ong, Catrin E. Moore, Katrina J. Charles, Dinesh Mondal, Bruno Silvester Lopes, Shahana Parveen

**Affiliations:** 1 Laboratory of Environmental Health, Laboratory Sciences and Services Division, International Centre for Diarrhoeal Disease Research, Dhaka 1212, Bangladesh; 2 Burnett School of Biomedical Sciences, University of Central Florida, Orlando, FL 32816, USA; 3 Emerging Infections, Infectious Diseases Division, International Centre for Diarrhoeal Disease Research, Dhaka 1212, Bangladesh; 4 School of Geography and the Environment, University of Oxford, Oxford OX1 2JD, UK; 5 Big Data Institute, Nuffield Department of Medicine, University of Oxford, Oxford OX1 2JD, UK; 6 Department of Medical Microbiology, School of Medicine, Medical Sciences and Nutrition, University of Aberdeen, Aberdeen AB25 2ZD, UK; 7 National Horizons Centre, Teesside University, Darlington DL1 1HG, UK

**Keywords:** antibiotic resistance, extended spectrum β-lactamase producing *Klebsiella pneumoniae* (ESBL-KP), carbapenem-resistant *Klebsiella pneumoniae* (CRKP), healthcare facilities, biofilm

## Abstract

The emergence of virulent extended spectrum β-lactamase producing *Klebsiella pneumoniae* (ESBL-KP) including carbapenem-resistant *Klebsiella pneumoniae* (CRKP) in hospital-acquired infections has resulted in significant morbidity and mortality worldwide. We investigated the antibiotic resistance and virulence factors associated with ESBL-KP and CRKP in tertiary care hospitals in Bangladesh and explored their ability to form biofilm. A total of 67 ESBL-KP were isolated from 285 *Klebsiella pneumoniae* isolates from environmental and patient samples from January 2019 to April 2019. For ESBL-KP isolates, molecular typing was carried out using enterobacterial repetitive intergenic consensus polymerase chain reaction (ERIC-PCR), antibiotic susceptibility testing, PCR for virulence and drug-resistant genes, and biofilm assays were also performed. All 67 isolates were multidrug-resistant (MDR) to different antibiotics at high levels and 42 isolates were also carbapenem-resistant. The most common β-lactam resistance gene was *bla*_CTX-M-1_ (91%), followed by *bla*_TEM_ (76.1%), *bla*_SHV_ (68.7%), *bla*_OXA-1_ (29.9%), *bla*_GES_ (14.9%), *bla*_CTX-M-9_ (11.9%), and *bla*_CTX-M-2_ (4.5%). The carbapenemase genes *bla*_KPC_ (55.2%), *bla*_IMP_ (28.4%), *bla*_VIM_ (14.9%), *bla*_NDM-1_ (13.4%), and *bla*_OXA-48_ (10.4%) and virulence-associated genes such as *fimH* (71.6%), *ugeF* (58.2%), *wabG* (56.7%), *ureA* (47.8%) and *kfuBC* (28.4%) were also detected. About 96.2% of the environmental and 100% of the patient isolates were able to form biofilms. ERIC-PCR-based genotyping and hierarchical clustering of *K. pneumoniae* isolates revealed an association between environmental and patient samples, indicating clonal association with possible transmission of antimicrobial resistance genes. Our findings can help in improving patient care and infection control, and the development of public health policies related to hospital-acquired infections.

## 1. Introduction

*Klebsiella pneumoniae* is linked to community and hospital-acquired infections and shows high resistance to antibiotics. The present disease load in Bangladesh is unknown; however, it is higher in susceptible individuals, including newborns and the elderly. The unregulated and irrational use of antibiotics can often lengthen treatment, cause treatment failure and lead to increased disease burden [1,2]. *K. pneumoniae* is a well-known hospital-acquired pathogen responsible for infections of the urinary tract system, lungs, and blood having been recently linked to pyogenic liver abscesses (PLA), which can be aggravated by endophthalmitis, meningitis, necrotizing fasciitis, and prostatic abscess [3,4].

In 2017, the World Health Organization designated Enterobacterales that produce ESBLs, including *K. pneumoniae*, as pathogens of critical priority for antibiotic research and development [5]. In *K. pneumoniae*, there are two significant types of antibiotic resistance. One process involves the production of ESBLs, which provide resistance to cephalosporins and monobactams. The production of carbapenemases by *K. pneumoniae* is another method of resistance, making the bacterium resistant to practically all the β-lactams, including carbapenems. CRKP is responsible for a variety of hospital-acquired infections around the world, showing resistance to all β-lactam antibiotics including a variety of other vital therapeutic drugs [3,4,6]. The most prevalent enzymes detected are Ambler class A KPCs, class B metallo-lactamases (VIM, IMP, and NDM-1), and class D OXA-type enzymes (OXA-48-like). *K. pneumoniae* can also develop carbapenem resistance by the overexpression of efflux pumps or by mutations in porins genes. The overexpression of ESBLs or AmpC-lactamases is also responsible for carbapenem resistance [7].

The capsule and lipopolysaccharide are the major virulence factors in *K. pneumoniae*. The *rmpA* gene, known as a positive regulator of extracapsular polysaccharide synthesis, is associated with highly virulent *K. pneumoniae* [8]. In addition, iron-scavenging systems and adhesion through fimbriae and non-fimbriae are also responsible for virulence. *K. pneumoniae* is a pathogenic bacterium with active iron acquisition mechanisms, and using an iron chelator siderophore, it takes up protein-bound iron out of its host cell [9,10]. It uses the surrounding capsular polysaccharide to protect itself from the bactericidal action of serum and to hinder phagocytosis [11,12]. In *K. pneumoniae*, there are eight O-antigen serotypes and 77 K-antigen serotypes [13,14,15,16], with serotypes K1, K2, K4, and K5 being the most virulent in a mouse model and potentially causing severe infections in humans and animals [17]. Moreover, *K. pneumoniae* serotype K2, the second most common serotype after serotype K1 responsible for *K. pneumoniae* associated liver abscess, is commonly observed in community-acquired pneumonia, predominating in human infections [18]. FimH is an adhesive subunit of type 1 fimbriae present in different bacteria and is shown to be critical for the ability of *K. pneumoniae* to cause UTI in a murine model [19] and ureA is responsible for gastrointestinal tract colonization [20].

*Klebsiella* spp. has been known to produce biofilms both inside the body and on abiotic surfaces like plastic, indwelling equipment, and invasive devices like catheters [21]. For bacteria, the capacity to build a biofilm is a crucial colonization factor. Biofilms are aggregated bacterial cells enclosed within a polysaccharide matrix [22] that may withstand host defense mechanisms and antibiotic exposure [23]. Compared to planktonic (free-living) cells, growth in biofilms increases (1000 fold) assisting the survival and persistence of bacterial populations in hospital environments, thereby increasing the risk of hospital-acquired infections [24,25,26]. Biofilm-associated infections are hard to treat, even with proper antibiotic therapies, because of slow access of the antimicrobial agent through the biofilm matrix and the changed growth rate of the organism in the biofilm [21,27]. The high resistance to antibiotics among biofilms was attributed to the presence of an extracellular polymeric constituent, which forms a physical barrier that inhibits drug penetration.

Molecular typing is vital for understanding the genetic links between different bacterial strains, as well as identifying outbreaks and possible hospital-acquired infection origins. It is especially essential for hospital-acquired *K. pneumoniae* infections and for enhancing outbreak management. Though various techniques, that is, pulsed-field gel electrophoresis (PFGE), enterobacterial repetitive intergenic consensus-polymerase chain reaction (ERIC-PCR), randomly amplified polymorphic DNA (RAPD), whole genome sequencing (WGS), and core genome multilocus sequence typing (cgMLST) have been used to type *K. pneumoniae* [28,29,30,31], ERIC-PCR is effectively employed to genotype *K. pneumoniae* isolates from the various origin, and it is considered one of the most efficient, comparatively easy, and cost-effective techniques [28,32]. This also provides an excellent basis for understanding the molecular epidemiology of *K. pneumoniae*, particularly in resource-limited countries.

In this investigation, we looked for virulent genes in *K. pneumoniae* that were ESBL positive (ESBL-KP) and resistant to carbapenem antibiotics (CRKP) in both patients and hospital surrounding (environmental) samples in three Bangladeshi tertiary care hospitals. These ESBL-KP and CRKP isolates were also analyzed for their biofilm-forming properties and their association between antibiotic resistance and biofilm-forming capacity.

## 2. Results

### 2.1. Distribution of ESBL-KP and CRKP among the Hospitals

From the patients and environmental samples surrounding their beds, a total of 285 *K. pneumoniae* isolates were identified and among them 67 were ESBL-producer. From these 67 isolates, 41.8% (28/67) were recovered from swabs taken from the floor, 26.9% (18/67) from the pillow on the bed, 20.9% (14/67) from nasal-throat samples of patients, 9% (6/67) from caregivers’ hands, and 1.5% (1/67) from wound pus samples. Among the 67 isolates, 62.7% (42/67) of the isolates were carbapenem-resistant. These isolates were found mainly on the floor at 25.4% (17/67), bed pillows at 20.9% (14/67), and nasal-throat at 16.4% (11/67). None of the isolates from the caregivers’ hand or pus sample was carbapenem-resistant.

In our study, we observed 23.5% (67/285) ESBL-KP isolates in all three studied hospitals. Among the 67 ESBL-KP isolates, 52 were isolated from the surrounding environment and 15 were isolated from patients as carriage samples. The distribution of CRKP in our studied hospitals was diverse. Using microbiological culture 90% (9/10) of the isolates from Rangpur medical college hospital; 85.7% (18/21) of the isolates from Rajshahi medical college hospital and 41.7% (15/36) of the isolates from Faridpur medical college hospital were resistant to carbapenems. The distribution of ESBL-KP and CRKP among different sampling points of various hospitals was illustrated in Table 1.

### 2.2. Antibiotic Resistance Patterns of the Isolates

**Figure 1:**
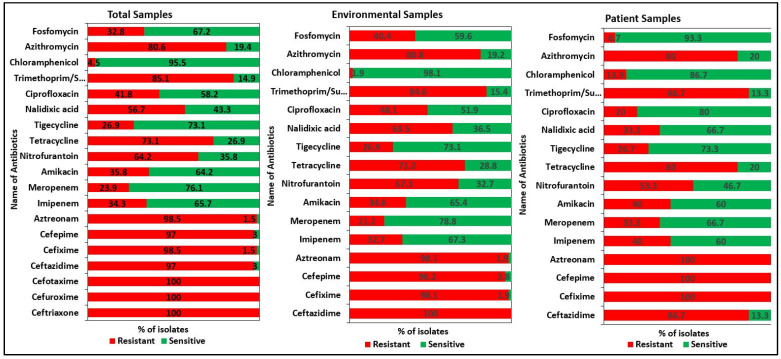
Antibiotic resistance patterns of the *K. pneumoniae* isolates. All the isolates were tested against 19 antibiotics from 14 different therapeutic classes. All the isolates were classified as multi drug-resistant MDR and extensively drug-resistant (XDR) but none of them as pan drug-resistant (PDR).

The antibiotic susceptibility patterns of the 67 isolates were evaluated using 19 antibiotics from 14 different therapeutic classes. All the isolates were classified as MDR and 10.5% (7/67) of them as extensively drug-resistant (XDR) but none of them as pan drug-resistant (PDR) [33]. Second- and third-generation (2G and 3G) cephalosporin (ceftriaxone, cefuroxime, and cefotaxime) resistance was found in all 67 isolates. About 98.5% (66/67) of the isolates showed resistance to 3G cephalosporin (cefixime) and monobactam (aztreonam), followed by 97% (65/67) to 3G (ceftazidime) and 4G cephalosporin (cefepime), 85.1% (57/67) to sulfamethoxazole-trimethoprim, 80.6% (54/67) to macrolides (azithromycin), 73.1% (49/67) to tetracycline, 64.2% (43/67) to nitrofuran derivatives (nitrofurantoin), 41.8% (28/67) to fluoroquinolones (ciprofloxacin), 34.3% (23/67) and 28.4% (19/67) to imipenem and meropenem, respectively. However, most of the *K. pneumoniae* strains were sensitive to chloramphenicol with only 4.5% (3/67) resistance (Figure 1). 

**Figure 2:**
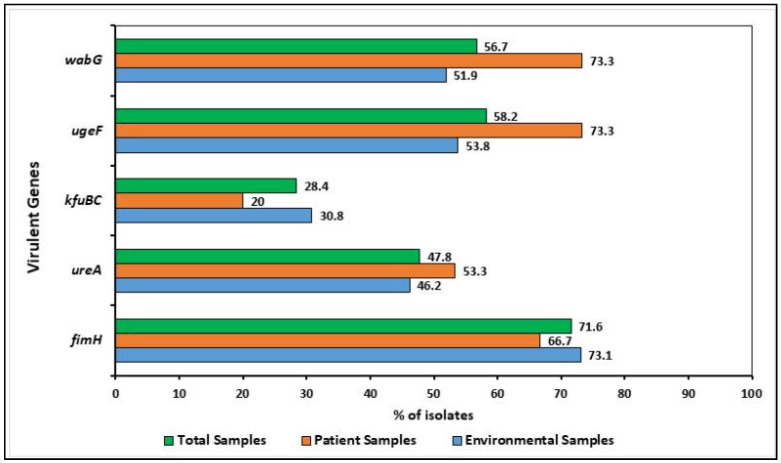
Distribution of virulence determinants (i.e., *fimH*, *wabG*, *ugeF*, *ureA*, and *kfuBC*) among the *K. pneumoniae* isolates. The virulent genes were commonly present in both environmental and patient isolates.

Our study showed noticeable differences in antibiotic susceptibility between environmental and patient isolates (Figure 2). All 52 environmental isolates showed resistance to the 2G and 3G cephalosporins (cefotaxime, cefuroxime, ceftriaxone, and ceftazidime). In the case of 15 patient isolates, 100% resistance was found for the 2G, 3G, and 4G cephalosporins and monobactams (cefotaxime, cefuroxime, ceftriaxone, cefepime, cefixime, and aztreonam). For imipenem and meropenem, 30.8% (16/52) and 23.1% (12/52) resistance was observed in environmental isolates, respectively. Patient isolates were more resistant (46.6%, 7/15) to both imipenem and meropenem antibiotics. The antimicrobial resistance pattern of all the isolates is listed in Table S3.

### 2.3. Detection of Virulence and Resistance Genes

The distribution of five virulence genes is shown in Figure 2. Of the 67 isolates, 62 harbored at least one virulence gene, whereas five isolates carried all five tested virulent genes. The virulence and virulence-associated genes *fimH*, *wabG*, *ugeF*, *ureA*, and *kfubc* were commonly present in both environmental and patient isolates and 71.6% (48/67) *fimH*, 56.7% (38/67) *wabG*, 58.2% (39/67) *ugeF*, 47.8% (32/67) *ureA*, and 28.4% (19/67) *kfuBC* were detected in the sampled isolates, respectively. Among the 52 environmental isolates, 47 harbored at least one virulence gene, whereas 55.3% (26/47), 38.3% (18/47), and 6.4% (3/47) genes were detected from the floor samples, bed pillow, and caregivers’ hand, respectively. For patient samples, all of them harbored at least one virulence gene.

**Figure 3:**
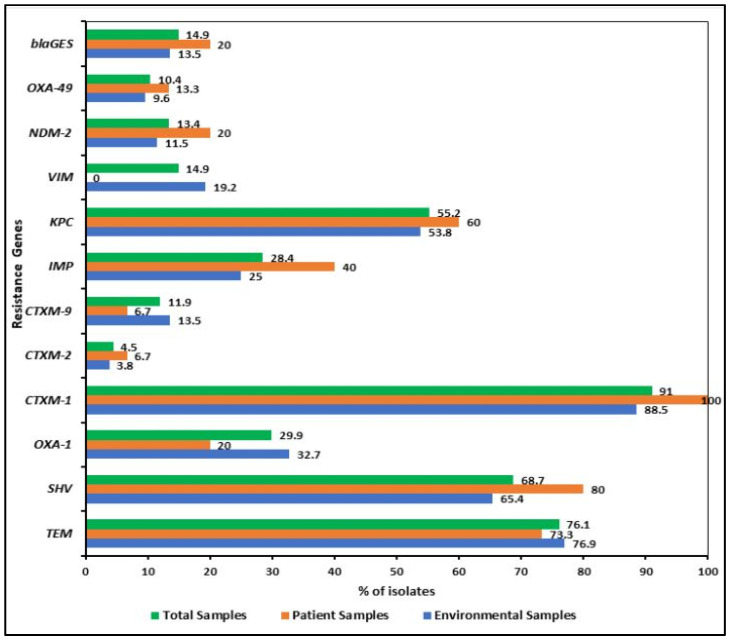
Distribution of β-lactamase (i.e., *bla*_TEM_, *bla*_SHV_, *bla*_OXA-1_, *bla*_CTX-M-1_, *bla*_CTX-M-2_ or *bla*_CTX-M-9,_ *bla*_GES_) including carbapenemase (*bla*_VIM_, *bla*_IMP_, and *bla*_KPC_ and *bla*_OXA-48_) genes among the *K. pneumoniae* isolates. Three or four broad-spectrum β-lactamase genes were frequently found in the isolates but the distribution of carbapenemase genes varied among patient and environmental isolates.

**Figure 4:**
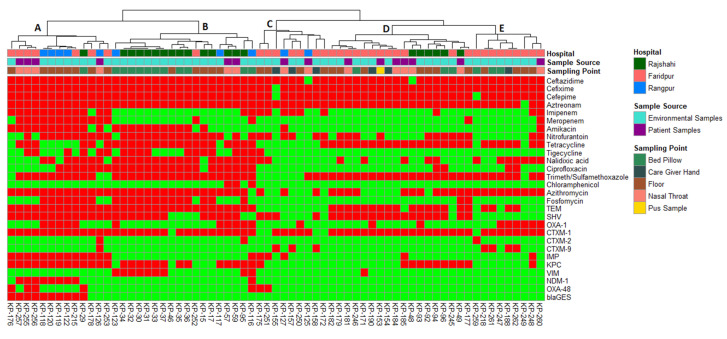
Heatmap and hierarchical clustering of *K. pneumoniae* isolates according to their phenotypic (antibiotic resistance) and genotypic (antibiotic resistance genes) profile of variables showing differences between isolates. Red color represented presence and green color represented the absence of resistance or gene. Left of the heatmap is a color representation of the different sources (patient in blue and environmental in red) and sampling points from those sources. Hierarchical clustering was performed using Wald’s method and a binary distance matrix. Letters (A–E) designate the 5 main clusters described in the text.

PCR was carried out for all the *K. pneumoniae* isolates to detect ESBL and carbapenemase genes. Three or four broad-spectrum β-lactamase genes (*bla*_TEM_, *bla*_SHV_, *bla*_OXA-1_, *bla*_CTX-M-1_, *bla*_CTX-M-2_ or *bla*_CTX-M-9_) were frequently found in the isolates. Among the β-lactamase genes, the most frequent one encountered was *bla*_CTX-M-1_, which was found in 91% (61/67) of the study isolates, followed by *bla*_TEM_ in 76.1% (51/67), *bla*_SHV_ in 68.7% (46/67) in the total ESBL positive isolates (Figure 3). The targeted carbapenemase genes were tested in all isolates where 55.2% (37/67) contained *bla*_KPC_ and *bla*_IMP_ was detected in 28.4% (19/67) of strains (Figure 3). The class A β-lactamase genes encoding ESBLs, *bla*_CTX-M-1_, *bla*_TEM_, and *bla*_SHV_, were found in 88.5% (46/52), 76.9% (40/52) and 65.4% (34/52) of environmental isolates, respectively. Interestingly, *bla*_CTX-M-1_ (100%, 15/15) and *bla*_SHV_ (80%, 12/15) genes were more prevalent in patient isolates than in patients’ surrounding environmental ones. All the isolates, except one (KP-172), harbored *bla*_TEM_, *bla*_SHV_, *bla*_OXA-1_, *bla*_CTX-M_ genes (Figure 4).

**Figure 5:**
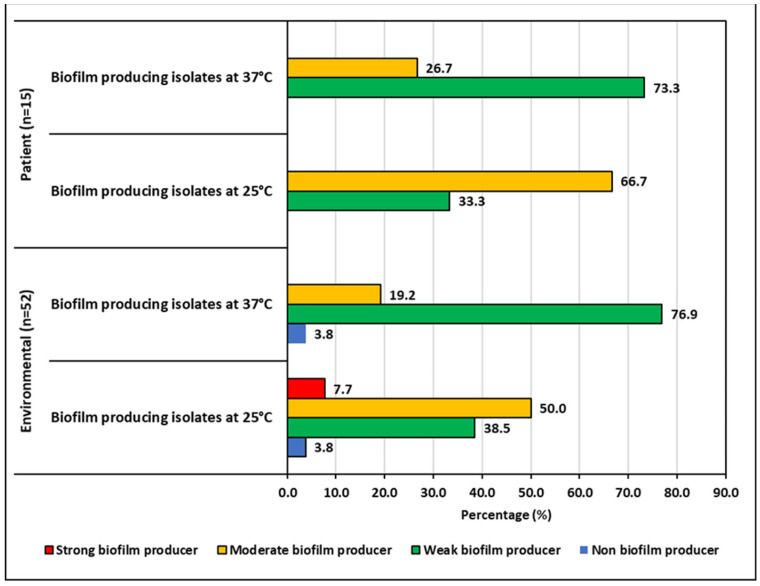
Biofilm producing capacity of *K. pneumoniae* isolates.

The carbapenemase genes indicated their distribution varied among patient and environmental isolates. *bla*_KPC_-, *bla*_IMP_-, *bla*_NDM-1_-, and *bla*_OXA-48_-like genes were more prevalent in patient isolates than the environmental isolates (Figure 5). Surprisingly, no patient isolates possessed carbapenemase gene *bla*_VIM_, present in 19.2% (10/52) of the environmental isolates. Table S2 has a detailed description of the β-lactamases including carbapenemases identified in the 67 KP isolates.

### 2.4. Biofilm Forming Ability of the ESBL-KP and CRKP Isolates

The ability to produce biofilms was temperature-dependent for both the environmental and patient isolates. After 48 h of incubation, the biofilm production increased at 25 °C and reduced at 37 °C. Environmental isolates produced less biofilm than the patient isolates at both temperatures. Half of the environmental isolates showed moderate biofilm production at 25 °C, whereas it was 66.67% for patient isolates. The number of isolates for different categories of biofilm producers can be seen in Figure 5.

### 2.5. Molecular Typing of the Isolates

**Figure 6:**
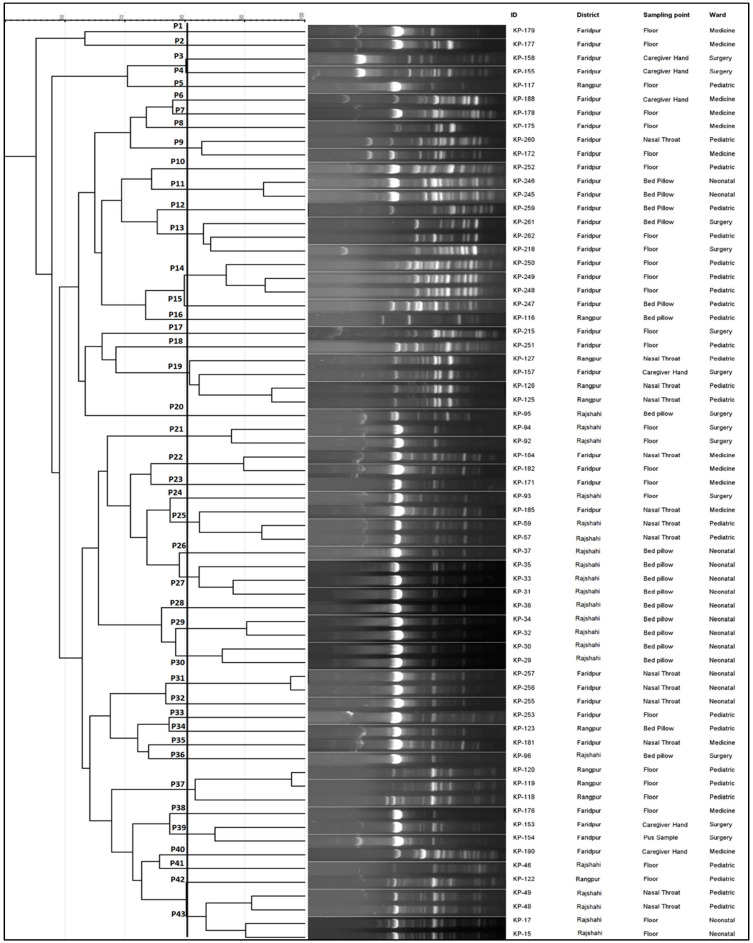
ERIC-PCR molecular fingerprint profiles of *K. pneumoniae* isolates from patients and hospital environment along with their representative ID, geological location, sampling point, and location. In the dendrogram, 43 distinct patterns (P1-P43) of *K. pneumoniae* isolates with similarity > 80% were observed. The isolates produced 4–19 amplicons ranging from ~140 to ~1300 bp, where ~300, ~410, and ~680 bp were common among the isolates.

Enterobacterial Repetitive Intergenic Consensus Polymerase Chain Reaction (ERIC-PCR) was used to explore the relationship between the carbapenemase and ESBL-producing pathogenic *K. pneumoniae*. Figure 6 depicts the genetic profiling of isolates using ERIC-PCR fingerprinting. According to the dendrogram, ERIC analyses revealed 43 distinct patterns (P1–P43) of *K. pneumoniae* isolates with similarity >80%. The isolates produced 4–19 amplicons ranging from ~140 to ~1300 bp, where ~300, ~410 and ~680 bp were common among the isolates. We found that the maximum number of four isolates belonged to both P19 and P43 patterns, suggesting their genetic similarity, and a single isolate in each of 28 different patterns. The isolates (KP-119 and KP-120) obtained from the pediatric floor in the same hospital showed the highest similarity and identical antibiotic resistance gene profiles, suggesting that those isolates are from the same clonal lineage (Figure 4 and Figure 6). Likewise, two isolates (KP-256 and KP-257) obtained from the nasal-throat of the neonatal ward of the same hospital also showed similar genetic profiles.

### 2.6. Associations between Sample Source and Phenotypic and Genotypic Traits

**Figure 7:**
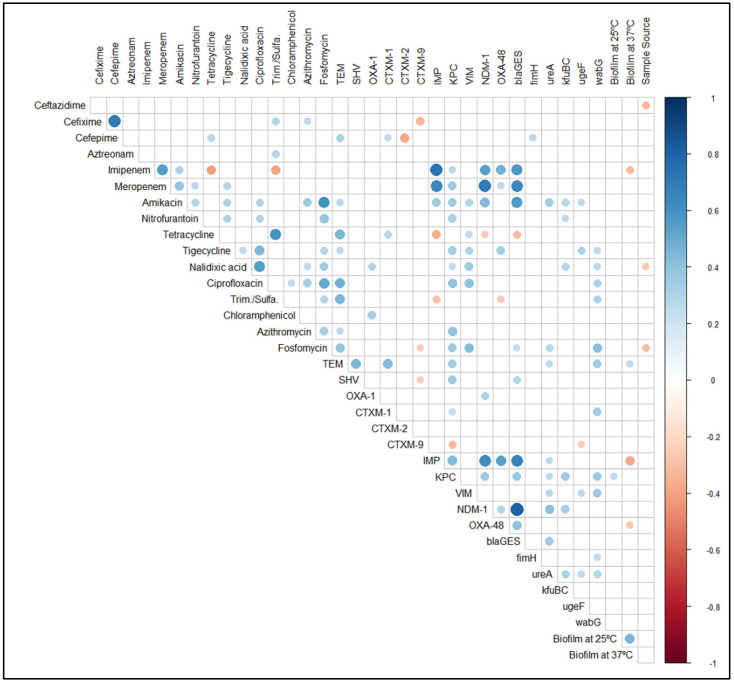
Correlation matrix of phenotypic (antibiotic resistance and biofilm formation ability) and genotypic (antibiotic resistance genes and virulence genes) features shows significant correlations. White spaces are not significantly correlated. Blue circles indicated a significant positive correlation and red circles show a significant negative correlation. The size and strength of color represent the numerical value of the Phi correlation coefficient. The correlation matrix shows only significant (*p* < 0.05) associations, as assessed by the Chi-square test.

**Figure 8:**
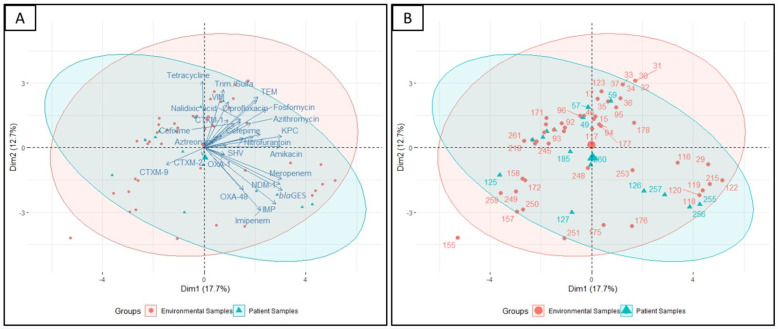
Principal component analysis performed on variables showing differences. (**A**) Visualization of the isolates encompassed in 95% confidence intervals grouping based on the source of the isolate (environmental or patient) and (**B**) labeling of the individual isolates from the same analysis.

Hierarchical clustering (along with heatmap) (Figure 4), correlation matrix analysis (Figure 7), and principal component analysis (Figure 8A,B) were used to determine associations between the phenotypic and genotypic traits and the source of the isolates. The correlation coefficient and *p*-values obtained from correlation analysis showed very few positive relationships with the presence of β-lactamase genes and resistance to β-lactams. However, resistance to cefepime significantly correlates with the presence of the *bla*_TEM_ and *bla*_CTX-M-1_ genes (Figure 8, *p* < 0.05, r = 0.31 and 0.25, respectively). In the case of the carbapenem group of antibiotics, resistance to imipenem and meropenem were significantly correlated with the presence of carbapenemase genes- *bla*_KPC_, *bla*_IMP_, *bla*_NDM-1_, and *bla*_OXA-48_ like genes (Figure 8, *p* < 0.05).

Significant positive correlations of phenotypic antibiotic resistance indicated that co-occurrence of resistance was prevalent (Figure 8, *p* < 0.05). For instance, resistance to the fluoroquinolone group antibiotic, ciprofloxacin, was positively correlated with chloramphenicol, amikacin, fosfomycin, azithromycin, nitrofurantoin, nalidixic acid, and tigecycline (Figure 8, *p* < 0.05). Similarly, resistance to fosfomycin was positively correlated with resistance to the combination drug sulfamethoxazole-trimethoprim, amikacin, azithromycin, nitrofurantoin, nalidixic acid, ciprofloxacin and tigecycline (Figure 8, *p* < 0.05). Alongside the co-occurrence of resistance, we also found the coexistence of virulence genes and antibiotic resistance in both ESBL-KP and CRKP isolates. The presence of virulence genes was also examined where a significant positive correlation between type 1 fimbriae adhesive subunit *FimH* and the resistance to cefepime (Figure 8, *p* < 0.05, r = 0.28) was observed. Likewise, the presence of *ureA* gene was positively correlated with the resistance to amikacin, and fosfomycin (Figure 8, *p* < 0.05). Among the virulence genes, the presence of *wabG* gene was positively correlated with the resistance to the highest numbers of antibiotics, namely tigecycline, nalidixic acid, ciprofloxacin, sulfamethoxazole-trimethoprim and fosfomycin (Figure 8, *p* < 0.05).

There was no significant correlation between antimicrobial resistance and biofilm formation in ESBL-KP and CRKP isolates. However, we observed an association between biofilm formation and resistance to some specific antibiotics. All the isolates resistant to imipenem, meropenem, and amikacin showed the ability to form biofilm at different levels. In imipenem-resistant isolates, 56.5%, 39.1%, and 4.3% were categorized as weak, moderate, and strong biofilm formers, respectively. A similar percentage of biofilm formers was also found for meropenem-resistant isolates (Table 2). For amikacin, 66.7% of the resistant isolates were moderate biofilm formers and the rest were weak biofilm formers. In the same way, higher percentages of tetracycline-, ciprofloxacin-, and fosfomycin-resistant isolates showed weak to moderate biofilm-forming ability. Similar results were also observed in the case of other antibiotics including the β-lactams (Table 2).

The principal component analyses showed similar relations of β-lactam antibiotic cefepime with the β-lactamase genes *bla*_TEM_ and association of imipenem with the *bla*_OXA-48_-like carbapenemase gene (Figure 8A). Moreover, the isolates that showed resistance to cefepime were also resistant to fosfomycin, azithromycin, aztreonam, and cefixime. Isolates that harbored *bla*_CTX-M-2_ and *bla*_CTX-M-9_ β-lactamase genes did not show resistance to cefepime. The presence of β-lactamase genes of *bla*_TEM_ and *bla*_CTX-M-1_ were also visualized to be in association with ciprofloxacin and nalidixic acid resistance (Figure 8A).

Hierarchical clustering analysis allowed the segregation of the 67 *K. pneumoniae* isolates according to their phenotypic (antibiotic resistance profiles) and genotypic (resistance gene profiles) traits (Figure 4). Interestingly, a separation of *K. pneumoniae* isolated from environmental sources (Figure 4, Cluster B) and patient sources (Figure 5, Cluster D) was observed. Hierarchical clustering segregated the isolates into five main clusters. Cluster A included 9 *K. pneumoniae* isolates from the environment samples (eight of them from the floor and the remaining one from the bed pillow) and four from patient origins (all of them from the nasal-throat). Most of the isolates of cluster A were from Rangpur and Faridpur hospitals. Three isolates of Rangpur hospital (KP-118, KP-119, and KP-120) also clustered together in ERIC PCR. Of interest in this cluster, an environmental isolate (KP-176, floor) clustered together with three patient isolates (KP-257, KP-255, and KP-256; nasal-throat) of the same hospital and the clustering was visible in PCA as well (Figure 8B). KP- 257 and KP-256 clustered together in ERIC PCR also. Similarly, the clustering of patient isolate KP-126 with the environmental isolates KP-118, KP-120, KP-119, and KP-122 of Rajshahi hospital was also reflected in PCA (Figure 8B). Here, one of the large clusters, cluster B, consisted of 16 isolates from the environment (11 from the bed pillow and 5 from the floor) and two isolates from patient sources (nasal-throat). Among them, 12 isolates from Rajshahi hospital were clustered together. Corresponding to cluster A, here patient isolate KP-59 clusters together with the environment isolates (KP-30, KP-31, KP-32, KP-33, KP-34, KP-35, and KP-37) of the same hospital in PCA (Figure 8B). Cluster C in Figure 4 was relatively smaller and confined, with seven environmental isolates (four from the floor and three from the caregiver’s hand) and two patient isolates (nasal-throat) mostly from Faridpur hospital. Another large cluster (Figure 4, Cluster D) contained 12 *K. pneumoniae* isolates from the environment (seven from the floor, three from the bed pillow, and two from the caregiver’s hand) and six isolates from patient samples (five from the nasal-throat and the remaining from pus sample). In this cluster, isolates from Faridpur and Rajshahi hospitals were clustered together. Remarkably, a patient isolate (KP-184) and an environmental isolate from a caregiver’s hand (KP-154) from the same hospital were clustered together, which was also observed in PCA. While being from different sources in the same hospital, these two isolates demonstrated the same phenotype and genotype. Likewise, to cluster A and B in Figure 4, patient isolate KP-48 clustered together with the environment isolates (KP-92, KP-93, KP-94, and KP-96) of the same hospital which is also reflected in PCA. The remaining smaller cluster (Figure 4, Cluster E) had eight isolates from the environment (four from the floor, three from the bed pillow, and one from the caregiver’s hand) and one from the patient origin (nasal-throat) and all of them from the same hospital. Notably, in cluster E, the clustering of patient isolate KP-260 and environmental isolate KP-248 was also reproduced in PCA (Figure 8B).

## 3. Discussion

*K. pneumoniae* is responsible for various multidrug-resistant infections, and any transmission between the environment and patients has yet to be proven [34,35] in Low to Middle Income Countries (LMICs). The current study assessed the molecular characterization of antibiotic resistance genes, biofilm-forming capacity, and virulence factors associated with ESBL-KP and CRKP isolated from hospitalized patients and hospital environments. In a recent study in Bangladesh, 30% of the 3G cephalosporin-resistant *K. pneumoniae* present in clinical samples among hospitalized patients was identified as carbapenem-resistant [36]. Similar results were also reported in other studies for CRKP and other important hospital-acquired pathogens [37,38], which are in agreement with our findings.

The inappropriate use of antimicrobials in hospitals leads to the high prevalence of resistance in *K. pneumoniae* [39]. Multidrug-resistant, ESBL-KP are major causes of hospital-acquired infections which are difficult to treat, particularly in LMICs [34,35]. All the *K. pneumoniae* isolates in our study showed resistance to ceftriaxone, cefuroxime, and cefotaxime. Cephalosporins and penicillins are widely used antibiotics in Bangladesh [40] and all of our tested isolates were resistant to these antibiotics. A high prevalence of resistance was also observed against cefixime, aztreonam, cefepime, ceftazidime, trimethoprim-sulfamethoxazole, azithromycin, tetracycline, nitrofurantoin, and nalidixic acid. These are in line with the prevalence of resistance observed globally among hospital-acquired infections in Nepal, China, and Pakistan [41,42,43]. In our study, all the ESBL-KP isolates were MDR showed high resistance to the major antibiotics either alone or in combination, such as β-lactams (including 3G and 4G cephalosporins, monobactams, carbapenems), aminoglycosides, quinolones, and glycylcycline. There is limited information available for such a high frequency of MDR *K. pneumoniae* from patient samples and hospital environmental samples in Bangladesh, although a high incidence of MDR *K. pneumoniae* has been observed before from clinical samples around the world [34,41]. In the current study, 30.8% and 23.1% of the environmental isolates were imipenem and meropenem resistant respectively, for patient isolates, the resistance patterns peaked at 46.7%, which is an alarming rise in the carbapenem resistance scenario among clinical isolates of Bangladesh compared to the past few years [44,45,46].

The evading mechanisms of *K. pneumoniae* against carbapenem, are very crucial to investigate. In the present study, we examined the prevalence of ESBL genes (*bla*_CTX-M-1_, *bla*_CTX-M-2_ and *bla*_CTX-M-9_, *bla*_TEM_, *bla*_SHV_, *bla*_GES_, and *bla*_OXA-1_) and carbapenemase genes (*bla*_IMP_, *bla*_KPC_, *bla*_VIM_, *bla*_NDM-1_, and *bla*_OXA-48_ like genes) in *K. pneumoniae* from patient and environmental isolates. Among the ESBL-genes, *bla*_CTX-M_, *bla*_TEM_, and *bla*_SHV_ are the primary genes associated with most of the *K. pneumoniae* infections [47,48], which corresponds to the findings of our research. In most countries, the *bla*_CTX-M_ type of ESBL is the most prevalent, surpassing that of *bla*_SHV_ and *bla*_TEM_ ESBLs [49,50]. We also found *bla*_CTX-M-1_ to be the most frequent gene in the ESBL-positive *K. pneumoniae* isolates, followed by *bla*_TEM_ and *bla*_SHV_, which confer resistance to fluoroquinolone, aminoglycoside, and β-lactams [51]. Our result is a bit higher than the previous study with *bla*_CTX-M_ (51.4%), *bla*_TEM_ (49.4%), and *bla*_SHV_ (26.8%) in *K. pneumoniae* originated from clinical isolates in Bangladesh [45]. Carbapenems are the most commonly used antibiotics to treat severe infections caused by Enterobacterales that produce ESBLs [51,52]. The prevalence of *bla*_KPC_ in our study was 55.2%; with 88% of the CRKP isolates containing this gene, which is higher compared to the rates previously described in clinical samples from Bangladesh [44,53]. We also found that 14.9% of the isolates contained the *bla*_GES_ gene class, which behaves like carbapenemase or those that hydrolyze the extended-spectrum beta-lactam class of antibiotics. As we did not sequence these genes, we were unable to precisely identify the *blaGES* variants, some of which behave like carbapenemase (GES-2, -4, -5, -6) or those that hydrolyze the extended-spectrum beta-lactam class of antibiotics such as cephamycins and cephalosporins (GES-1, -10, -11, -12, -13) (Lopes *et. al*, unpublished data). Apart from the class A carbapenemases, KPC and GES, class B metallo-β-lactamases (MBLs) such as IMP, VIM, NDM, and class D carbapenem-hydrolyzing oxacillinase-48 (OXA-48) have also been the most often discovered carbapenemases in *K. pneumoniae* worldwide [20]. We examined a number of carbapenemase genes in our study and found a diverse prevalence of genes IMP, VIM, NDM-1, and OXA-48 [44], which is the first description of these genes found in hospital environmental samples in Bangladesh.

The findings of the virulence genes suggest that the *fimH*, *ureA*, *ugeF*, *kfuBC*, and *wabG* genes were frequently disseminated among patient and hospital environmental isolates [54,55]. The presence of these genes in hospital-associated environments could indicate that these isolates are pathogenic and provide a possible hospital-acquired risk to patients. The gene *fimH* encodes adhesins of type 1 fimbriae, which is responsible for attachment to the extracellular matrix, promotes colonization and biofilm formation, invasion, and pathogenicity [54,55,56], upon testing we found that 71.6% of the MDR *K. pneumoniae* contained this gene, indicating their potential ability to form biofilms. In addition to *fimH*, the gene responsible for the production of lipopolysaccharide (LPS), *wabG*, and *ugeF* genes were also present in more than half (56.7% and 58.2%) of the isolates, respectively [57,58]. The importance of these genes has been studied as their presence in *K. pneumoniae* indicates the strains are able to cause diseases such as urinary tract infection, pneumonia, and sepsis [59,60]. In our study, nearly half (47.8%) of strains contained the *ureA* gene, which helps in gastrointestinal (GI) tract colonization and has been associated with *K. pneumoniae* hospital-acquired infections [20]. We also found the *kfuBC* gene in 28.4% of the studied isolates, which has previously been found to be more abundant among invasive clinical strains such as those that arise from liver abscesses and are responsible for meningitis or endophthalmitis [4,61], explains the lower prevalence among environmental and patient strains.

*Klebsiella* spp. is known to form biofilm on biotic (e.g., epithelial surfaces) and abiotic surfaces, that is, on plastic, used in indwelling medical devices such as catheters and endotracheal tubing [62]. In the current study, we found that patient isolates were more likely to be weak to moderate biofilm formers than the environmental isolates, previously clinical samples have been described as producing high percentages of biofilm [63,64]. In our study, biofilm formation increased at 25 °C for both patient and environmental isolates. We postulate that this may be due to stress caused by the suboptimal temperature [64].

Interestingly, though no clear associations were observed between source types (patient or environmental) and antibiotic resistance or gene profile- *K. pneumoniae* from patient origins showed more resistance to β-lactams including carbapenems, quinolones, macrolides, and nitrofurantoin compared to environmental samples. The coexistence of virulence and antibiotic-resistant genes as revealed from our correlation analysis is in agreement with previous studies [65,66]. We found positive associations between biofilm production and specific antibiotics resistance, including imipenem, meropenem, amikacin, ciprofloxacin, tetracycline, and fosfomycin. Similar findings were also reported previously with associations between resistance to piperacillin-tazobactam and colistin correlating with biofilm formation in *K. pneumoniae* [67].

The association between ERIC-PCR genotype and antibiotic resistance of *K. pneumoniae* has been described previously from hospital isolates [34]. Because of variances in nucleotide sequences, pathogenic *K. pneumoniae* is very diverse [68]. For instance, isolates screened from the same sampling point and hospital, KP-118, KP-119, and KP-120, grouped in cluster A of hierarchical clustering, also represented similar genotypic patterns in ERIC. Similarly, KP-255-257; KP-31, KP-33, KP-35, KP-37, KP-92, and KP-94 also followed the trend with similar antibiotic-resistant determinants and genotypic patterns from the same hospital.

One of the limitations of the study was that we did not have the clinical information of the patients. Clinical information would have shed some light to understand the actual scenario of *K. pneumoniae* infections in the healthcare facilities of Bangladesh. Additional genes examining the efflux pump mediated resistant genes and capsule and type III encoding virulence genes would also have provided more discrimination and newer methods such as whole genome sequencing would have provided more insight into resistance and pathogenicity at the genome level.

## 4. Materials and Methods

### 4.1. Sample Collection and Isolation of K. pneumoniae

**Figure 9:**
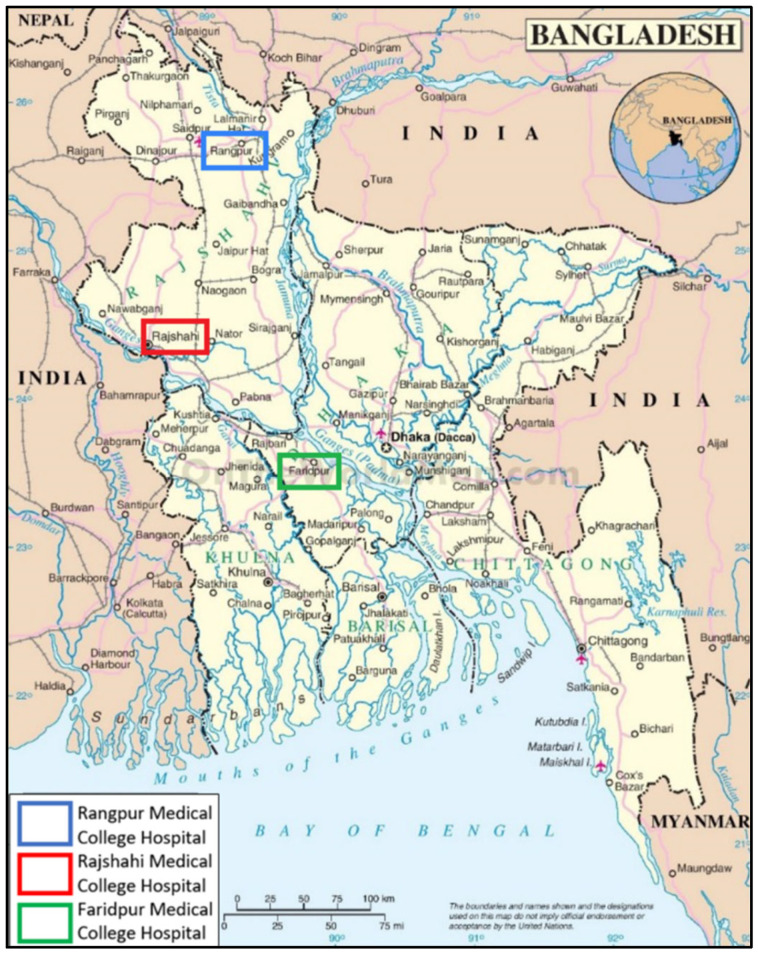
Geographical locations of the studied hospitals. Each hospital has several wards, including adult male and female medicine, surgery, pediatrics, and neonatal wards [69].

Over the course of four months, January 2019 to April 2019, samples were collected from three tertiary care medical college hospitals in Bangladesh: Faridpur, Rajshahi, and Rangpur. The geographical locations of these hospitals are shown in Figure 9.

Samples were taken from several wards within these hospitals, including adult male and female medicine, surgery, pediatrics, and neonatal wards. All swabs were transported to the Laboratory of Environmental Health, International Centre for Diarrhoeal Disease Research, Bangladesh (icddr,b), Mohakhali, Dhaka for all laboratory work. A convenient sampling procedure was performed on admitted patients and their immediate surroundings in the hospital to investigate the presence of antibiotic-resistant *K. pneumoniae*. The patient samples, including pus and nasal-throat swabs, were collected from convenient patients in the specified wards of the hospitals, together with the swabs from corresponding surfaces of the patient’s bed pillow, bedside rails, caregivers’ hands, and the floor (per 100 cm^2^) of that ward.

A sterile cotton swab was saturated with physiological saline, dabbed over the surface, and then transported to the icddr,b laboratory within 8–12 hours of collection in phosphate-buffered saline (PBS) while maintaining the cold chain (4–10 °C). A vortex mixer was used to homogenize the PBS containing swabs. The homogenized liquid was then spread onto a MacConkey Agar (BD-Difco, New Jersey, USA) plate and incubated at 37 °C for 24 h. Gram staining and indole testing were performed on suspected *K. pneumoniae* colonies on MacConkey agar, and the API 20E system was used to confirm the results (bioMerieux, Marcy-l’Étoile, France). Positive isolates were chosen for PCR using species-specific 16S rRNA primers [70]. Cultures were stocked in 30% glycerol-Lysogeny Broth (BD-Difco, New Jersey, USA) and stored at −80 °C for further investigation.

The dab inoculation method [71,72] was used to inoculate fresh single colonies of *K. pneumoniae* from the MacConkey agar plate on chromogenic ESBL and KPC (CHROMagar, Paris, France) plates. Metallic blue colonies on both chromogenic ESBL and KPC plates represent *K. pneumoniae* that can produce ESBL and are resistant to carbapenems [73]. The characteristics of the patient samples with respect to their environmental surroundings are described in the Table 3.

The isolates were evaluated for antimicrobial susceptibility to 19 antibiotics from 14 classes on Mueller–Hinton agar (BD-Difco, New Jersey, USA) following the Kirby–Bauer disk diffusion method in accordance with guidelines of the Clinical and Laboratory Standards Institute (CLSI) and European Committee on Antimicrobial Susceptibility Testing (EUCAST) [74,75,76,77]. For the result interpretation, we used the guideline of CLSI 2021 for all the antibiotics except tigecycline. For tigecycline, we used EUCAST 2021 guideline. The antibiotics used were, cefuroxime (30 μg), cefixime (5 µg), ceftriaxone (30 μg), ceftazidime (30 μg), cefotaxime (30 μg), cefepime (30 μg), aztreonam (30 μg), imipenem (10 μg), meropenem (10 μg), amikacin (30 μg), nitrofurantoin (300 μg), tetracycline (30 μg), tigecycline (15 μg), nalidixic acid (30 μg), ciprofloxacin (5 μg), trimethoprim-sulfamethoxazole (25 μg), chloramphenicol (30 μg), azithromycin (15 μg), and fosfomycin (50 μg). The clinical breakpoints provided by CLSI and EUCAST were used to classify isolates as susceptible or resistant and *E. coli* ATCC-25922 was used as a quality control strain. Intermediately resistant isolates were considered resistant in our investigation since we were looking at population-level resistance.

### 4.2. Detection of Virulence and Resistance Genes

The DNA was extracted from the isolates following the boiling lysis technique [78,79]. Multiplex PCRs using particular primers and conditions were used to screen all of the isolates, as previously described [80]. PCR for *kfuBC* (iron acquisition system-related gene), *fimH* (encoding type 1 fimbriae), *ugeF* (capsule-associated genes), *ureA* (first gene of urease gene cluster), and *wabG* (endotoxin-related genes) virulence genes was also performed as described earlier [17,32,81,82,83]. The PCR amplifications were carried out following the previously described protocol using the primers for ESBL encoding genes i.e., *bla*_TEM_/*bla*_SHV_/*bla*_OXA-1_, *bla*_CTX-M_ and carbapenemase encoding genes, that is, *bla*_VIM_, *bla*_IMP_, *bla*_KPC_ and *bla*_OXA-48_. PCR was also performed for *blaGES* genes (GES 1–9, 11 variants) and *blaNDM-1* gene [46,84]. Table S1 lists the primer sequences, annealing temperature, and amplicon sizes.

### 4.3. Molecular Typing of K. pneumoniae

ERIC-PCR was performed for molecular typing using the primer ERIC2 (5′-AAGTAAGTGACTGGGGTGAGCG-3′). 2 μL of DNA template were mixed with 12.5 μL of DreamTaq^TM^ Green PCR Master mix (Thermo Scientific, Vilnius, Lithuania), 1 μL of primer (10 pmol), and an adequate volume of sterile nuclease-free water to make a 25 μL reaction mixture. The PCR was carried out in a Bio-Rad T100™ Thermal Cycler (Bio-Rad, Hercules, CA, USA), with the following protocol: initial denaturation at 94 °C for 15 min, followed by 40 cycles of amplification. Denaturation at 95 °C for 1 min, primer annealing at 37 °C for 1 min, and primer extension at 72 °C for 1 min were used in each cycle. After the amplification cycles, samples were kept at 72 °C for 10 min to allow partially produced DNA to extend [78]. Then, 2% *w/v* agarose gel electrophoresis was used to visualize ERIC fragments, and the data were evaluated using GelJ v.2.0 software (Open source Java software) [83,85]. Invitrogen 1 kb plus ladder (Thermo Fisher Scientific, Waltham, MA, USA) was used to normalize the fragment patterns in GelJ v.2.0 software [27].

### 4.4. Biofilm Formation

Biofilm formation was detected using a quantitative adhesion method [86,87]. Each isolate was grown overnight in LB (Luria-Bertani) at 37 °C. Following that, 2 μL of bacterial suspension was injected into a sterile 96-well microtiter plate (Corning Life Sciences, Kennebunk, ME, USA) containing 198 μL of LB. In each run, LB broth was added as a negative control. It was then incubated for 48 h, statically at 25 °C and 37 °C. Each well was gently rinsed three times with 200 μL PBS to avoid disturbing the biofilm, then dried inverted at room temperature. The resultant biofilm mass was dyed with 50 μL of 0.1% crystal violet for 15 min. Again, the microtiter plate was cleansed three times with 200 μL of PBS and dried. Finally, the stained biofilm mass was solubilized by dissolving the wells in 200 μL of 5% isopropanol. A microplate reader set to 590 nm was used to measure optical density (OD). The mean OD was calculated after three separate experiments with triplicate of each isolate and negative control. The average OD of negative controls + 3 × SD (standard deviation) of negative controls was used to designate the OD cut-off (ODc). Isolate OD ≤ ODc was classified as a non-biofilm producer. On the other hand, the isolate was grouped as biofilm producers, with weak biofilm producers if ODc < OD ≤ 2 × ODc; moderate biofilm producers if 2 × ODc < OD ≤ 4 × ODc; and strong biofilm producers if OD > 4 × ODc [86,87].

### 4.5. Statistical Analysis

For statistical assessment, antibiotic resistance summaries along with harboring/lack of virulent and resistance genes were transformed to binary coding. Antibiotic sensitivity and resistance were denoted as 0 and 1, respectively. The harboring and lack of a given gene (for example, *bla*_TEM_) were likewise represented as 1 and 0, respectively. The open statistical program R (V: 4.1.1) was used to conduct the statistical analysis [7]. The ‘corr’ function was used to generate correlations for binary variables, and the ‘corr.test’ function was used to determine the significance using Pearson correlations and the (r) Phi coefficient. The ‘corrplot’ tool was used to illustrate significant correlations [36] and a heatmap, as well as hierarchical clustering, was prepared [88]. Ward’s approach was used to create distance matrices for hierarchical clustering [89]. For principal component analysis, the R packages ‘FactoMineR’ and ‘factoextra’ were utilized (PCA) [30,31].

## 5. Conclusions

Our study points out some worrisome findings in which *K. pneumoniae* present throughout the hospital environment is resistant to frequently used antibiotics to treat infections as well as last-resort medications for life-threatening illnesses. Additionally, our findings showed the presence of genetic determinants responsible for virulence and antibiotic resistance among the isolates, which might be transferable via conjugation and transposable genetic elements within the hospital environment. This is the first study assessing the presence of virulent and multidrug-resistant *K. pneumoniae* among both environmental and carriage samples in the hospital settings of Bangladesh. Most of the studied isolates produced biofilms when exposed to laboratory-induced stress, a feature that would be important in hospital-acquired infections originating from hospital surroundings and medical devices. The outcomes of this study also indicate an association between the presence of virulence genes, antibiotic resistance, and biofilm-forming capacity in *K. pneumoniae*. As a result, our findings should serve as a warning about the necessity for MDR *K. pneumoniae* prevention and control in Bangladeshi hospitals, and the need for rigorous infection prevention and control practices.

## Figures and Tables

**Table 1:** Distribution of ESBL producing and carbapenem-resistant *K. pneumoniae* isolates among different sampling points in different tertiary care medical college hospitals.

Hospital	Resistance	Sampling Point				
		Floor	Bed Pillow	Nasal Throat	Pus	Caregivers’ Hand
**Faridpur**	ESBL-KP	47.2%	13.9%	19.4%	2.8%	16.7%
		(17/36)	(5/36)	(7/36)	(1/36)	(6/36)
	CRKP	60.0%	6.7%	33.3%	0.0%	0.0%
		(10/15)	(1/15)	(4/15)		
**Rangpur**	ESBL-KP	50.0%	20.0%	30.0%	-	-
		(5/10)	(2/10)	(3/10)		
	CRKP	55.6%	22.2%	22.2%	-	-
		(5/9)	(2/9)	(2/9)		
**Rajshahi**	ESBL-KP	28.6%	52.4%	19.0%	-	-
		(6/21)	(11/21)	(4/21)		
	CRKP	16.7%	61.1%	22.2%	-	-
		(3/18)	(11/18)	(4/18)		

**Table 2:** Association between MDR *K. pneumoniae* isolates and their biofilm producing capacity.

Antibiotics	Number of Resistant Isolates	Biofilm 25 °C				Biofilm 37 °C		
		Non-Biofilm Producer	Weak Biofilm Producer	Moderate Biofilm Producer	Strong Biofilm Producer	Non-Biofilm Producer	Weak Biofilm Producer	Moderate Biofilm Producer
**Ceftriaxone**	67	2 (3%)	25 (37.3%)	36 (53.7%)	4 (6%)	2 (3%)	51 (76.1%)	14 (20.9%)
**Cefuroxime**	67	2 (3%)	25 (37.3%)	36 (53.7%)	4 (6%)	2 (3%)	51 (76.1%)	14 (20.9%)
**Ceftazidime**	65	2 (3.1%)	24 (36.9%)	35 (53.8%)	4 (6.2%)	2 (3.1%)	50 (76.9%)	13 (20%)
**Cefotaxime**	67	2 (3%)	25 (37.3%)	36 (53.7%)	4 (6%)	2 (3%)	51 (76.1%)	14 (20.9%)
**Cefixime**	66	2 (3%)	25 (37.9%)	36 (54.5%)	3 (4.5%)	2 (3%)	51 (77.3%)	13 (19.7%)
**Cefepime**	65	2 (3.1%)	25 (38.5%)	35 (53.8%)	3 (4.6%)	2 (3.1%)	50 (76.9%)	13 (20%)
**Aztreonam**	66	2 (3%)	24 (36.4%)	36 (54.5%)	4 (6.1%)	2 (3%)	50 (75.8%)	14 (21.2%)
**Imipenem**	23	0 (0%)	13 (56.5%)	9 (39.1%)	1 (4.3%)	1 (4.3%)	20 (87%)	2 (8.7%)
**Meropenem**	19	0 (0%)	11 (57.9%)	7 (36.8%)	1 (5.3%)	1 (5.3%)	16 (84.2%)	2 (10.5%)
**Amikacin**	24	0 (0%)	8 (33.3%)	16 (66.7%)	0 (0%)	0 (0%)	23 (95.8%)	1 (4.2%)
**Nitrofurantoin**	43	2 (4.7%)	14 (32.6%)	24 (55.8%)	3 (7%)	2 (4.7%)	32 (74.4%)	9 (20.9%)
**Tetracycline**	49	2 (4.1%)	16 (32.7%)	30 (61.2%)	1 (2%)	2 (4.1%)	37 (75.5%)	10 (20.4%)
**Tigecycline**	18	2 (11.1%)	5 (27.8%)	10 (55.6%)	1 (5.6%)	1 (5.6%)	13 (72.2%)	4 (22.2%)
**Nalidixic Acid**	38	2 (5.3%)	14 (36.8%)	21 (55.3%)	1 (2.6%)	2 (5.3%)	33 (86.8%)	3 (7.9%)
**Ciprofloxacin**	28	2 (7.1%)	7 (25%)	18 (64.3%)	1 (3.6%)	1 (3.6%)	23 (82.1%)	4 (14.3%)
**Trimethoprim/Sulfamethoxazole**	57	2 (3.5%)	19 (33.3%)	34 (59.6%)	2 (3.5%)	2 (3.5%)	44 (77.2%)	11 (19.3%)
**Chloramphenicol**	3	0 (0%)	0 (0%)	2 (66.7%)	1 (33.3%)	0 (0%)	1 (33.3%)	2 (66.7%)
**Azithromycin**	54	2 (3.7%)	20 (37%)	31 (57.4%)	1 (1.9%)	2 (3%)	51 (76.1%)	14 (20.9%)
**Fosfomycin**	22	1 (4.5%)	5 (22.7%)	16 (72.7%)	0 (0%)	2 (3%)	51 (76.1%)	14 (20.9%)

**Table 3:** Characteristics of the patients and environmental samples surrounding their beds in tertiary care hospitals in Bangladesh.

Isolate ID for Patient Sample	Hospital	Ward	Sampling Point	Corresponding Environmental Isolate ID	Corresponding Environmental Sampling Point
KP-48	Rajshahi	Pediatric	Nasal-Throat	KP-46	Floor
KP-49	Rajshahi	Pediatric	Nasal-Throat	KP-46	Floor
KP-57	Rajshahi	Pediatric	Nasal-Throat	KP-46	Floor
KP-59	Rajshahi	Pediatric	Nasal-Throat	KP-46	Floor
KP-125	Rangpur	Pediatric	Nasal-Throat	KP-116, KP-117, KP-118, KP-119, KP-120, KP-122, KP-123	Bed pillow, Floor
KP-126	Rangpur	Pediatric	Nasal-Throat	KP-116, KP-117, KP-118, KP-119, KP-120, KP-122, KP-123	Bed pillow, Floor
KP-127	Rangpur	Pediatric	Nasal-Throat	KP-116, KP-117, KP-118, KP-119, KP-120, KP-122, KP-123	Bed pillow, Floor
KP-154	Faridpur	Surgery	Pus Sample	KP-153, KP-155, KP-157, KP-158	Caregivers’ Hand
KP-181	Faridpur	Medicine	Nasal-Throat	KP-171, KP-172, KP-175, KP-176, KP-177, 178, KP-179	Floor
KP-184	Faridpur	Medicine	Nasal-Throat	KP-188, KP-189	Caregivers’ Hand
KP-185	Faridpur	Medicine	Nasal-Throat	KP-188, KP-189	Caregivers’ Hand
KP-255	Faridpur	Neonatal	Nasal-Throat	KP-245, KP-246	Bed pillow
KP-256	Faridpur	Neonatal	Nasal-Throat	KP-245, KP-246	Bed pillow
KP-257	Faridpur	Neonatal	Nasal-Throat	KP-245, KP-246	Bed pillow
KP-260	Faridpur	Pediatric	Nasal-Throat	KP-259, KP-262	Bed pillow, Floor

## Data Availability

Data is contained within the article and Supplementary Material.

## Supplementary Materials

The following supporting information can be downloaded at: https://www.mdpi.com/article/10.3390/ph15091116/s1.
