# Supplementary material for: Healthcare Facilities as Potential Reservoirs of Antimicrobial Resistant Klebsiella pneumoniae: An Emerging Concern to Public Health in Bangladesh
Source: Pharmaceuticals (Basel). 2022 Sep 7;15(9):1116. doi: 10.3390/ph15091116 (PMC9504507; doi:10.3390/ph15091116)
Supplement: Supplementary file 1 [file pharmaceuticals-15-01116-s001.zip › Supplementary Table_S1.pdf]

Supplementary material

Table S1: Primer sequences, annealing temperature, and amplicon sizes for antibiotic-resistant andvirulent genes

Gene Name		Primer Name	Gene Sequence	Size	Ref.
Species-specific	*16S rRNA*	16S rRNA F1 (NM3_F)	ATGTCGCAAGACCAGAGTGG	658	[70]
		16S rRNA R1 (NM3_R)	GCACAACCTCCAAATCGACA		
Antibiotic Genes	*bla*TEM	MultiTSO-T F	CATTTCCGTGTCGCCCTTATT	800	[80]
		MultiTSO-T R	CGTTCATCCATAGTTGCCTGA		
	*bla*SHV	MultiTSO-S F	AGCCGCTTGAGCAAATTAAA	713	
		MultiTSO-S R	ATCCCGCAGATAAATCACCA		
	*bla*OXA-1-like	MultiTSO-O F	GGCACCAGATTCAACTTTCAA	564	
		MultiTSO-O R	GACCCCAAGTTTCCTGTAAGT		
	*Bla*CTX-M-1	MultiCTXMGp1 F	TTAGGAARTGTGCCGCTGYA	688	
		MultiCTXMGp1-2 R	CGATATCGTTGGTGGTRCCAT		
	*Bla*CTX-M-2	MultiCTXMGp2 F	CGTTAACGGCACGATGA	404	
		MultiCTXMGp1-2 R	CGATATCGTTGGTGGTRCCAT		
	*Bla*CTX-M-9	MultiCTXMGp9 F	TCAAGCCTGCCGATCTGG	561	
		MultiCTXMGp9 R	TGATTCTCGCCGCTGAA		
	*bla*GES	MultiGES F	AGTCGGCTAGACCGGAAAG	399	
		MultiGES R	TTTGTCCGTGCTCAGGAT		
	*bla*OXA-48-like	Multi0XA-48 F	GCNGATCGCCCTCGATÏ	281	
		MultiOXA 48 R	GATTTGCTCCGTGGCCGAAA		
	*bla*IMP	MultilMP F	TTGACACTCCATTTACDG	139	
		MultiIMP R	GATYGAGAAITAAGCCACYCT		
	*bla*VIM	MultiVIM F	GATGGTGTTTGGTCGCATA	390	
		MultiVIM R	CGAATGCGCAGCACCAG		
	*bla*KPC	MultiKPC F	CATTCAAGGGCTTTCTTGCTGC	538	
		MultiKPC R	ACGACGGCATAGTCATTTGC		
	*bla*NDM-1	NDM-F	CTTCCAACGGTTTGATCGTC	625	[46]
		NDM-R	TAGTGCTCAGTGTCGGCATC		
Virulent Genes	*kfuBC*	kfu-F1179	GAA GTG ACG CTG TTT CTG GC	797	[81]
		kfu-R649	TTT CGT GTG GCC AGT GAC TC		
	fimH	fim-1	GCTCTGGCCGATACC ACCACGG	423	
		fim-2	GCGATAGATAACGTCGCCTGGA ACGG		
	uge	uge-F	GATCATCCGGTCTCCCTGTA	534	
		uge-R	TCTTCACGCCTTCCTTCACT		
	wabG	wabG-F	CGGACTGGCAGATCCATATC	683	
		wabG-R	ACCATCGGCCATTTGATAGA		
	ureA	ureA-F	GCTGACTTAAGAGAACGTTATG	337	
		ureA-R	GATCATGGCGCTACCTTA		
